# Evaluation of Intrascleral Lakes after Phaco-Viscocanalostomy using Anterior Segment Optical Coherence Tomography

**DOI:** 10.18502/jovr.v19i2.13228

**Published:** 2024-06-21

**Authors:** Saeed Shokoohi-Rad, Amir-reza Ansar, Abbas Vatandoost, Javad Firoozi

**Affiliations:** ^1^Eye Research Center, Mashhad University of Medical Sciences, Mashhad, Iran; ^2^Eye Research Center, The Five Senses Research Institute, Rassoul Akram Hospital, Iran University of Medical Sciences, Tehran, Iran

**Keywords:** Anterior Segment Optical Coherence Tomography, Intrascleral Lake, Phaco-Viscocanalostomy, Primary Open Angle Glaucoma

## Abstract

**Purpose:**

This study aimed to investigate the results of combined phacoemulsification and viscocanalostomy (phaco-VC) in a six-month follow-up and its relationship with intrascleral lake (IL) using anterior segment optical coherence tomography (AS-OCT) in patients with primary open-angle glaucoma (POAG).

**Methods:**

In total, 36 eyes with POAG eligible for phaco-VC were enrolled in this prospective observational study. All patients underwent AS-OCT evaluation and ophthalmologic examination including Goldman tonometry, cup–disc ratio assessment, best corrected visual acuity (BCVA) measurement, and antiglaucoma medication(s) prior to surgery and one, three, and six months after the surgery. The width, length, area, and circumference of the ILs were evaluated using AS-OCT at each follow-up.

**Results:**

A total of 36 eyes of 34 patients with POAG were investigated in this study. According to the results, the mean age of the patients was 70.09 ± 8.73 years, and the majority of the cases were male (*n* = 23; 63.9%). The mean preoperative intraocular pressure (IOP) was 20.11 ± 7.22 mmHg on 2.47 ± 1.1 medications, and the mean postoperative IOP reduced to 11.11 ± 2.58 mmHg on 0.11 medications, which was statistically significant (*P* < 0.001). ILs were detectable in all cases which resulted in a 100% qualified success rate. The reduction in the width, area, and circumference of the IL was significant during the six-month follow-up. The relationship between IOP changes and IL parameters on AS-OCT was not significant.

**Conclusion:**

This study evaluated the associations between IL changes and IOP reduction after phaco-VC. A six-month follow-up showed a notable reduction in the IL, but unexpectedly, IOP control did not decline. A reduction in IL diameter, when there is sufficient IOP control, indicates that there may be various IOP lowering mechanisms through VC other than the IL diameters. Further evaluation of VC focusing on long-term changes in IL and Schlemm's canal diameter is necessary to explain the precise mechanisms of lowering the IOP.

##  INTRODUCTION

Glaucoma is the second cause of vision loss and its most prevalent subtype, namely primary open-angle glaucoma (POAG), affects more than 57 million people worldwide, and imposes an enormous burden on healthcare systems and causes a great inconvenience for patients.^[1,2,3]^ Although trabeculectomy, as the gold standard surgical treatment of POAG, accompanies considerable benefits in decreasing intraocular pressure (IOP), non-penetrating glaucoma surgery (NPGS) is associated with more safety and lower postoperative complications than opening the anterior chamber including hypotony, choroidal detachment, and endophthalmitis.^[4,5,6,7]^

The two most prevalent types of these non-penetrating glaucoma surgeries are viscocanalostomy (VC) and deep sclerectomy (DS).^[8]^ VC was firstly described by Stegmann.^[9]^ In this procedure, viscoelastic agents are injected meticulously into the Schlemm's canal to dilate and enhance the passage of aqueous.^[9]^ VC is also capable of lowering IOP without the formation of a filtering bleb, thereby circumventing possible bleb-associated complications.^[6]^ The exact mechanism of all these surgeries is not well-understood. It seems that bypassing the high-resistance juxtacanalicular structures causes easier percolation of aqueous via trabeculo-Descemet's membrane (TDM). Aqueous humor accumulates in the intrascleral space (lake) which then egresses through the uveoscleral pathway.^[10,11,13]^

Multiple authors proposed that maintaining the intrascleral lake (IL) is in favor of better long-term outcomes.^[4,7,14,15,16,17]^ As the IL is in the deep scleral layer, its evaluation using a slit-lamp is not accurate; therefore, numerous studies have focused on evaluating the structure of the blebs and ILs after performing DS using ultrasound biomicroscopy (UMB) or anterior segment optical coherence tomography (AS-OCT).^[8,11,12,14,15,16,17,18,19,20]^

AS-OCT is a competent alternative to UMB in evaluating bleb and deeper scleral structures because of its higher resolution, faster acquisition time, and non-contact method.^[21,22]^ The CASIA SS-1000 OCT (Tomey, Nagoya, Japan) is a Fourier-domain, swept-source OCT (SS-OCT) designed, specifically for imaging the anterior segment. This system achieves high-resolution imaging of 10 μ m (axial) and 30 μ m (transverse) and high-speed scanning of 30,000 A-scans per second. With a substantial improvement in scan speed, the anterior chamber angles can be imaged at 360º in 128 cross-sections (each with 512 A-scans) in about 2.4 s.^[23]^

Studies showed that AS-OCT findings of IL and bleb morphology could be associated with surgical success rate and IOP control.^[8]^ To the best of our knowledge, no study has evaluated the morphology of IL after VC using AS-OCT. The primary outcome of this study was to clarify the characteristics of IL after phacoemulsification and viscocanalostomy (phaco-VC) and its changes in a short-time follow-up. This study also looked at the associations of the IL parameters with IOP control, and its clinical application in predicting the success rate.

##  METHODS

This prospective non-randomized observational study was performed at Khatam-Al-Anbia Eye Hospital, a tertiary referral eye center affiliated to Mashhad University of Medical Sciences, Iran. Consecutive patients with advanced POAG, aged 50 to 80 years, and no history of ophthalmic surgery and no other ocular issues other than glaucoma and cataract were recruited from May 2018 to September 2020.

All patients had thorough ophthalmic evaluation, which included testing for visual acuity, slit-lamp examination, gonioscopy, IOP measurement using a Goldmann applanation tonometer, and fundoscopy to assess the optic disc. The diagnosis of POAG was made based on the patient's medical history, findings on gonioscopy, and evidence of visual field defects on perimetry (Humphrey Perimeter Analyzer, Zeiss AG, Germany) and the presence of corresponding loss of the retinal nerve fiber layer revealed on OCT.

Advanced POAG patients with poor IOP control despite maximal medical treatment, with or without visually significant cataracts, were enrolled in the study. Additionally, surgery was performed on patients who were unable to tolerate topical glaucoma treatments. These POAG patients were candidates for combined phacoemulsification and VC in this study.

Patients who had either primary or secondary closed-angle glaucoma were not included in the study. Additionally, patients who had intraoperative complications like trabeculo-Descemet membrane rupture or postoperative issues and those unwilling to continue the follow-ups were excluded. All surgeries were performed by an experienced glaucoma surgeon (SSR).

The complete and qualified success evaluation was according to the following parameters: Complete success was defined as IOP of ≤ 18 mm Hg without the use of anti-glaucoma medication, and qualified success was defined as IOP ≤ 18 mm Hg with the administration of an anti-glaucoma drug.

### Surgical Technique

**Standard phacoemulsification and intraocular lens (IOL) implantation were performed through a temporal clear corneal incision in an area other than the VC site. In all cases, a one-piece hydrophobic acrylic IOL was implanted in the bag.**

VC consisted of opening the conjunctiva and Tenon's capsule and creating a 5 × 5-mm limbus-based superficial scleral flap. A deeper scleral flap measuring about 4 × 4 mm with the maximum possible thickness was dissected. In the next step, the roof of the Schlemm's canal was removed and continued onto the cornea to reach the TDM. Then, a delicate cannula was introduced into the entrance of Schlemm's canal in the left and right directions, and high-viscosity sodium hyaluronate was injected gently into it for 4 to 6 mm. The deeper scleral flap was then excised (DS), and the superficial flap was sutured securely using 11-0 polyester fiber sutures. No antifibrotic agent was used at the time of the surgery or postoperatively.

All patients received chloramphenicol eye drops (ChlobioticⓇ 0.5%, Sina Daru Pharmaceutical Co., Iran) for two weeks and betamethasone eye drops (BetasonateⓇ – Betamethasone 0.1%, Sina Daru Pharmaceutical Co., Iran) for six to eight weeks after surgery. The corticosteroid eye drop was administered every 2 hr in the first week; then it was decreased to six times per day for three weeks, followed by three times per day for four weeks.

### OCT Procedure and Examination

Examinations included tonometry with the Haag-Streit Goldmann device and measurement of the cup-to-disc ratio (CDR) and checking the best-corrected visual acuity (BCVA) which were all performed prior to surgery and then at each subsequent visit. Surgical procedures and measurements of CDR before and after surgery were performed by a glaucoma sub-specialist. OCT analysis of the anterior segment and the IL were performed on the same day by a trained optometrist using the CASIA SS-1000 OCT system (Tomey, Nagoya, Japan).

**Figure 1:**
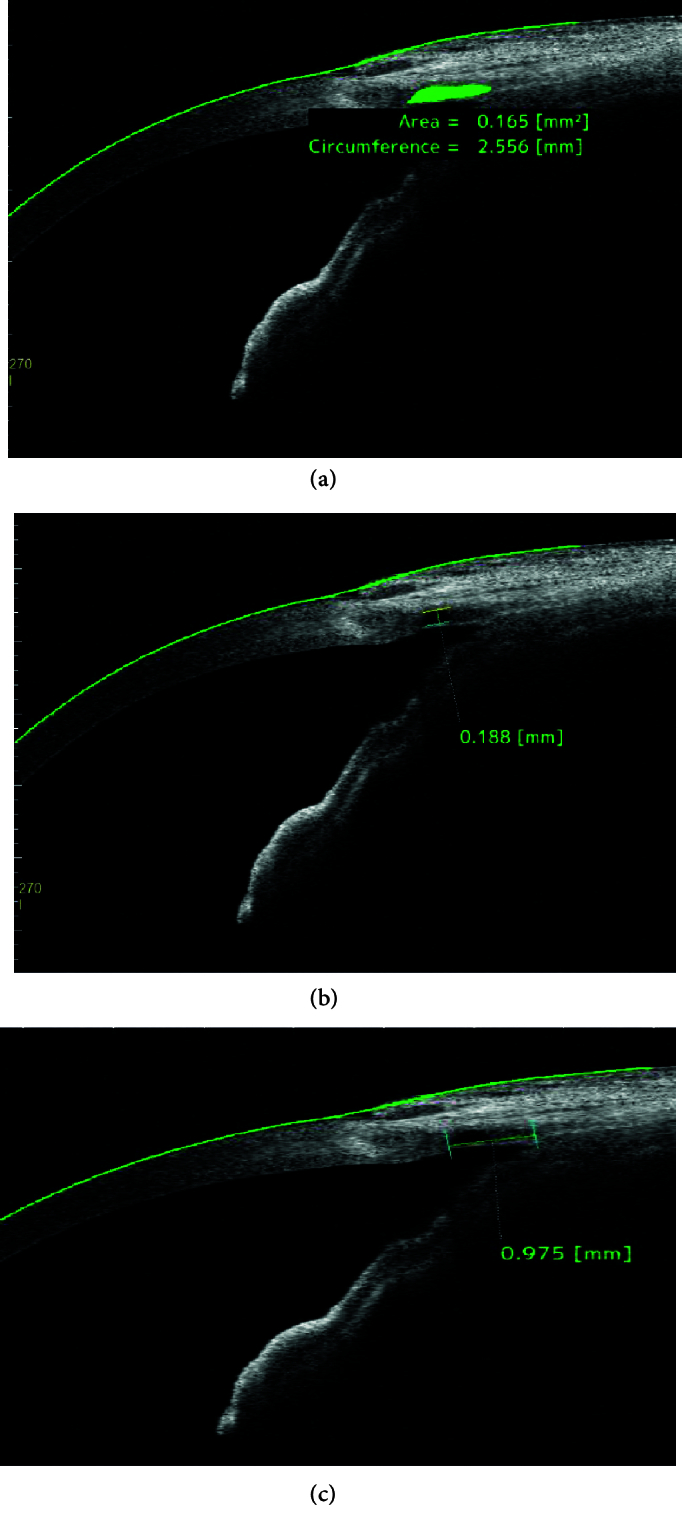
(a) Area and circumference of the intrascleral lake. (b) Intrascleral lake height. (c) Intrascleral lake length.

For this imaging, the patient looked down at a 30º angle at the horizontal plane. The operator retracted the upper lid gently to expose the upper bulbar conjunctiva as much as possible without putting pressure on the globe. Then, high-resolution scanning was performed at 45-, 90-, and 135º angles. With the two-dimensional image recorded by the device, the maximum IL sizes in each scan were measured manually using the OCT caliper tool and automatically calculated by the device software. Parameters examined in AS-OCT included the presence of a filtering bleb, the maximum height and anterior-posterior length of the IL, area, and circumference of the IL. The area and circumference of the IL were measured automatically by a built-in software after the margins of the hypoechoic space were marked manually. Figure 1 shows the area, circumference, length, and height of the IL. We defined the area of the IL as the hypo-echo space occupied by the IL inside the sclera and the circumference as the perimeter occupied by the IL (in the largest dimensions) [Figure 1a]. Moreover, the height of the IL was the vertical distance between two lines parallel to the cornea and sclera at the maximum possible or maximum measured position [Figure 1b].

The posterior–anterior length of the IL was defined as the distance between the first reflex signals from the sclera at the longest length [Figure 1c].

### Statistical Analysis 

This investigation was carried out as a pilot study on 34 patients because we were unable to find any comparable studies. The Kolmogorov–Smirnov test was used to check the normality. In addition, the Wilcoxon test examined the mean of the variables at the first, third, and sixth-month follow-ups. The Spearman correlation coefficient investigated the correlation between IL dimensions and IOP. The Mann–Whitney test was used for two independent groups.

In this study, the level of statistical significance was 0.05. All statistical analyses were performed using the SPSS program for Windows, version 25 (IBM SPSS Statistics, IBM Corporation, Chicago, IL, USA).

The study protocol adhered to the tenets of the Declaration of Helsinki. All participants provided written informed consent before enrollment, and the ethical aspects of the study were approved by the Regional Committee on Medical Ethics at Mashhad University of Medical Sciences, Mashhad, Iran (IR.MUMS.MEDICAL.REC.1397.321).

##  RESULTS

### Baseline Data 

In total, 36 eyes of 34 patients who had POAG and underwent phaco-VC by a single surgeon were included in this study. The majority of patients (*n* = 23; 63.9%) were male, and the mean age of the participants was 70.09 ± 8.73 years. The patient's characteristics are shown in Table 1.

Patients who had surgery-related complications, such as TDM rupture and iris incarceration, were excluded from the study. Five patients were excluded from the final analysis due to TDM ruptures. None of the patients underwent an additional procedure postoperatively, such as suturolysis, needling, or goniopuncture during the six months of follow-up.

### Surgical Outcomes 

**Figure 2:**
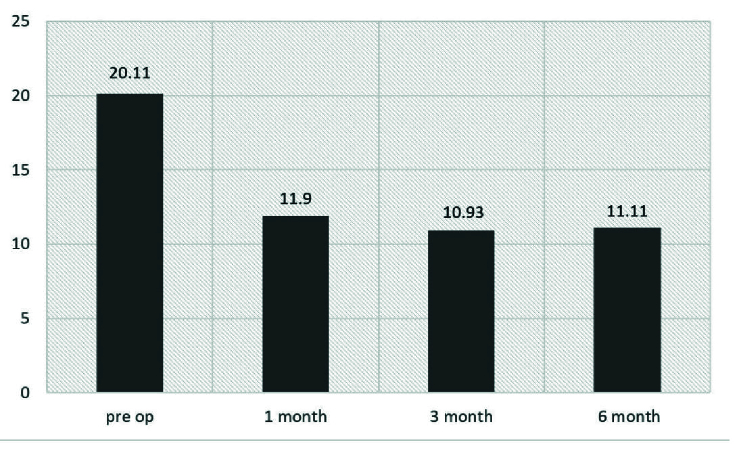
Intraocular pressures (mmHg) of the eyes before and after Phaco-VC.

The mean preoperative IOP of 20.11 ± 7.22 mm Hg decreased significantly to 11.9 ± 3.88 mm Hg at the first month, 10.93 ± 2.26mm Hg at the third month, and 11.11 ± 2.58 mm Hg at the sixth month (*P* < 0.001) [Figure 2]. One patient experienced an IOP spike (IOP = 28) in the first follow-up in which antiglaucoma medications started.

**Figure 3:**
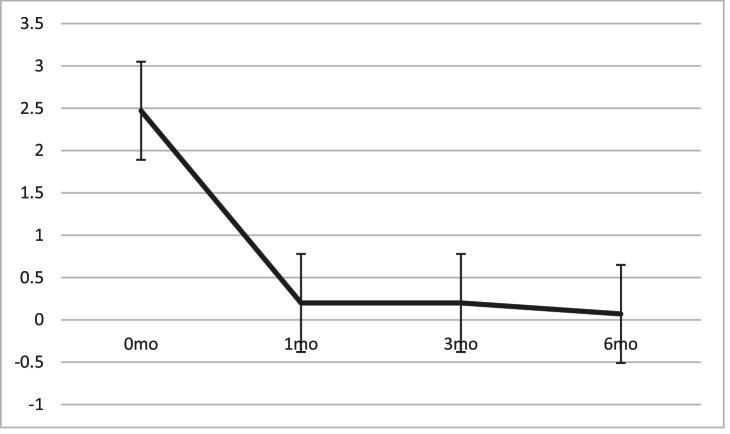
Number of antiglaucoma medications before and after Phaco-VC.

The preoperative number of antiglaucoma medications of 2.47 ± 1.1 statistically significantly decreased to 0.2 ± 0.55, 0.2 ± 0.61, and 0.11 ± 0.46 at the first, third, and sixth-month follow-ups, respectively (*P* < 0.001) [Figure 3].

**Figure 4:**
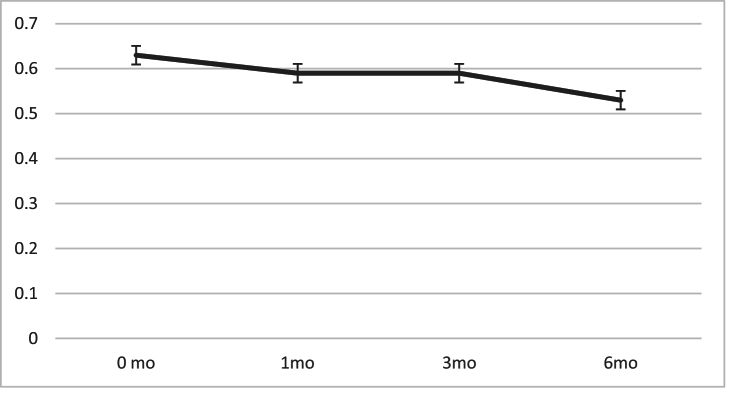
Best-corrected visual acuity (BCVA) changes at six-month follow-ups.

The mean BCVA before surgery was 0.63 ± 0.54 logMAR which improved to 0.59 ± 0.56 logMAR at the first follow-up examination (*P* = 0.542) and 0.55 ± 0.53 logMAR (*P* = 0.445) three months after the operation. In the last follow-up, patients' BCVA improved to 0.5 ± 0.57, which was almost significant compared to the preoperative BCVA (*P* = 0.056) [Figure 4].

The mean preoperative CDR was 0.83 ± 0.15 and measured clinically 0.83 ± 0.15, 0.83 ± 0.15, and 0.83 ± 0.15 at months one, three, and six, respectively. Comparisons between the pre-operation and follow-up groups did not reveal any statistically significant differences (*P* > 0.999). In this study, 34 of 36 (94.0%) eyes had an IOP < 18 mm Hg without the administration of medications at the end of a six-month follow-up and were categorized as a complete success. Additionally, two of them (6%) had an IOP < 18 with medications at the end of the study. Overall, a qualified success was achieved in all 36 eyes.

### Anterior Segment Optical Coherence Tomography Findings

**Figure 5:**
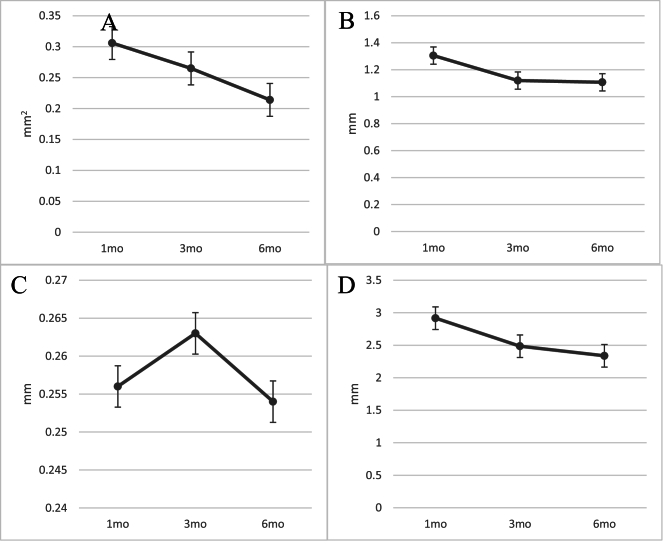
Postoperative changes of intrascleral lake's parameters. Area of the lake (A). Using the Wilcoxon Signed Ranks Test, significant difference (*P* = 0.001) is seen between the first-month findings (0.306 ± 0.374 mm^2^) and the sixth-month results (0.214 ± 0.238 mm^2^). (B) The lake's length. The first-month results (1.305 ± 0.906 mm) and the sixth-month results (1.107 ± 0.692 mm; *P* = 0.004) show a significant difference. (C) The lake's height. The first-month results (0.256 ± 0.148 mm) and the sixth-month results (0.254 ± 0.235 mm; *P* = 0.102) did not differ significantly. (D) The lake's circumference. Using the Wilcoxon Signed Ranks Test, there is a significant difference (*P* < 0.001) between the first-month findings (2.916 ± 2.01 mm) and the sixth-month data (2.337 ± 1.592 mm).

Figure 5 shows the typical AS-OCT changes of the IL parameters at the first, third, and sixth-month postoperative follow-ups. The mean IL area of 0.306 ± 0.374 mm^2^ in the first month decreased to 0.214 ± 0.238 mm^2^ in the sixth month (*P* = 0.001, using repeated measures ANOVA) [Figure 5]. The length of the lake decreased from 1.305 ± 0.906 mm in the first month to 1.107 ± 0.692 mm in the sixth month (*P* = 0.004). Further findings are shown in detail in Figure 5.

Table 2 shows the correlation between the changes of IL parameters (length, height, area, and circumference) and the percentage of IOP reduction during the follow-ups. According to the findings, the association between the changes in length, height, area, and circumference of the ILs, and the IOP change was not significant. In other words, a decrease in the size of IL did not lead to an elevated IOP in follow-ups.

The associations between IOP and AS-OCT parameters are described in Table 3. We examined the parameters that can predict better IOP control in the sixth-month follow-up. According to this table, there is no significant correlation between the IOP in first and six months with the findings in the AS-OCT.

**Table 1:** Description of demographic variables


**Numbers**	**36**
**Age**	
Mean ± SD (yr)	70.09 ± 8.73
Range	58–80
**Gender**	
Male	13 (36.1)
Female	23 (63.9)
**Laterality**	
OS	18 (50)
OD	18 (50)
**Preoperative BCVA **	
Mean ± SD (logMAR)	0.63 ± 0.54
Median	0.61
**Preoperative IOP**	
Mean ± SD (mmHg)	20.11 ± 7.22
Range	12-38
**Preoperative anti-glaucomatous medications**	
Mean ± SD	2.47 ± 1.1
	
	
white<bcol>2</ecol>IOP, intraocular pressure

**Table 2:** Correlation coefficients between IL and IOP changes at the six-month follow-up


	**IOP diff 1-6**
Length diff 1-6 ${\;}^{*}$	Spearman's R *P*-value	–0.219 0.245
Height diff 1-6	Spearman's R *P*-value	–0.133 0.490
Area diff 1-6	Spearman's R *P*-value	0.006 0.976
Circumference diff 1-6	Spearman's R *P*-value	–0.073 0.702
	
	
${\;}^{*}$ Changes in the Intrascleral Lake's length, height, area, circumference, and IOP, respectively, between the first and six-month follow-ups are represented by the terms Length diff, Height diff, Area diff, Circumference diff, and IOP diff.		

**Table 3:** Correlation of IOP at the first- and sixth-month follow-ups with IL parameters on AS-OCT


	<@		**Six-month IOP**	**First-month IOP**
First-month intrascleral lake parameters	Extent	Correlation coefficient	–0.103	0.031
	*P*-value	0.590	0.871
	Height	Correlation coefficient	–0.132	0.060
	*P*-value	0.494	0.757
	Circumference	Correlation coefficient	–0.070	0.061
	*P*-value	0.712	0.748
	Area	Correlation coefficient	–0.168	0.023
	*P*-value	0.374	0.904
Sixth-month intrascleral lake parameters	Extent	Correlation coefficient	–0.060	0.168
	*P*-value	0.752	0.375
	Height	Correlation coefficient	–0.077	0.096
	*P*-value	0.685	0.613
	Circumference	Correlation coefficient	0.012	0.184
	*P*-value	0.948	0.331
	Area	Correlation coefficient	–0.028	0.157
	*P*-value	0.884	0.409
	
	
AS-OCT, anterior segment optical coherence tomography; IL, intrascleral lake; IOP, intraocular pressure				

##  DISCUSSION

Complete and qualified success in glaucoma surgeries is defined according to the postoperative IOP decrease and the requirement for antiglaucoma medication administration. Although efficient IOP reduction in the post-operation visits is the most determining factor for the surgical outcome, surgeons are always encouraged to seek some visual clues on examination in concordance with effective IOP control. Since the observation of an elevated bleb after trabeculectomy predicts a desirable long-term outcome,^[8]^ the visualization of the deep filtering structures using AS-OCT may yield finding predicting parameters that are not usually visible through a slit-lamp examination.

The success of VC depends on many factors since multiple mechanisms play roles in lowering IOP, such as canaloplasty and unroofing Schlemm's canal. To the best of our knowledge, there are no similar studies designed to evaluate morphologic findings after VC on AS-OCT, however, it seems that the stability of IL is also influential in the long-term outcomes of NPGS.^[8]^

We found IL visible in AS-OCT in all 36 cases until the end of all follow-up periods. The IL length changes in the third-month follow-up (*P* = 0.02) and the sixth-month follow-up (*P* = 0.004) were significant, compared to the first-month follow-up. Changes in the IL circumference were significant in the follow-up of the third month, compared to the first month (*P* = 0.007) and in the follow-up of the sixth month, compared to the third month (*P* < 0.001). Regarding the area of the IL, the comparison of the follow-up of the third month to the first month (*P* = 0.004) and the sixth month to the third month (*P* = 0.001) shows a significant difference, while the changes in IL height in the third and sixth-month follow-up periods were not significant compared to the first month. These findings are in agreement with the results of previous studies that stated a decrease in the size of IL after DS with implants^[24,25]^ and after combined phaco-VC^[26]^ using UBM.

Although we observed a downward trend in the IL diameters that reflects the role of wound healing and the fibrosis process, IOP levels in this period were on a plateau even though the IL size was decreasing and no significant correlation was found between IOP and IL diameters. A study by Romero et al in POAG patients undergoing DS showed a correlation between IOP decline and IL height in AS-OCT.^[17]^ Buenaga et al found a moderate inverse correlation between IL height in AS-OCT and IOP measurements after DS with Esnoper and Aquaflow implants.^[18]^ Furthermore, other studies showed an inverse relationship between IL height in AS-OCT and IOP after DS with intraoperative 5-fluorouracil^[27]^ or with collagen implants^[15]^. Furthermore, Konstantopoulos et al claimed that the height of the bleb or IL correlates with the success rate of filtering procedures.^[1]^

These studies did not conclude the VC procedure, and their findings contradict our results as we could not realize an association between IOP and IL height. As there appears to be no exact study to compare with, this result could be a confounding effect of using antiglaucoma drops or may highlight the additional effects of VC, when compared to the DS procedure. Decreases in the size of the IL in the presence of an adequate IOP control represent the presence of other complementary lowering IOP mechanisms in VC other than the influence of IL diameters. We emphasize the importance of unroofing the Schlemm's canal and experiencing alternations in Schlemm's canal diameters for better IOP control. In support of this idea, a morphological evaluation after canaloplasty and DS using AS-OCT showed a close relationship between structural changes of the IL and IOP and an inverse relationship of IOP with the area, length, width, and height of the intrascleral bleb and the thickness of the TDM.^[16]^ Cheng et al also showed that maintaining the IL with implants does not determine a better IOP control.^[28]^

Park et al evaluated IL changes after phaco-VC using UBM. They reported a decrease in IL diameter and concomitant reduction in the magnitude of IOP control, and a detectable IL in 67% of cases after a 12-month follow-up, whereas we visualized the IL in all 36 cases after six months of follow-up which could be related to the high resolution of AS-OCT. We also observed a plateau in IOP control after surgery that contradicts their findings. These studies performed phaco-VC and used antiglaucoma medications occasionally in the postoperative follow-ups. We think this observation of a plateau in the readings of IOP after surgery in our study which contradicted the findings of Park et al could be related to the exclusion of complicated surgeries in our study.

In our study, the results of statistical analysis showed that at the sixth-month follow-up, BCVA (*P* = 0.056), number of post-operation medications (*P* < 0.001), and Goldman IOP (*P* < 0.001) had a statistically significant difference, when compared to preoperative data. Moreover, six months after surgery, 100% of patients encompassed qualified success, and 94% of the cases had complete success with a 35% reduction in IOP at month six, which implies it is a competent surgical technique for lowering IOP as previous studies showed.^[5,6]^ Because of the advanced stage of glaucoma in this study, the changes between preoperative and postoperative BCVA were not noticeable, and the primary goal of cataract surgery was to enhance the overall effect of VC^[29]^ rather than specifically improving visual acuity. One of our patients experienced an IOP of 28 mmHg in the first-month follow-up (two weeks after surgery) that decreased to < 18 mmHg after the initiation of antiglaucoma medication and cessation of the topical corticosteroid that could induce an elevated IOP.

AS-OCT is a helpful optical device to visualize subconjunctival and intrascleral changes after NPGS. Many mechanisms of action have been proposed for VC. Understanding the exact under-structural alternations after performing VC can illuminate the cardinal role of each strategy in achieving better IOP control and clarifies the need to focus on those methods that promises success without aimless excessive manipulations. This study evaluated the association between IL changes and IOP reduction after VC in POAG patients. ILs were detectable in all cases which resulted in a 100% qualified success rate. Shrinkage of ILs at the sixth-month follow-up was significant; however, surprisingly, IOP control was not compromised. We attributed it to the adjutant intraoperative alteration in the Schlemm's canal anatomy. Further evaluation on VC while focusing on long-term changes in the IL and Schlemm's canal, especially by a 3D AS-OCT is needed. Furthermore, it is recommended to perform larger studies to investigate the minimum size of IL necessary for achieving long-term success and determining the predictive values of early failure.

The study population in our study was small and also the follow-up time was short which can lead to misinterpretation of the results. Moreover, it is better to conduct a randomized controlled study and compare DS with VC to delineate the role of every adjuvant component for better IOP control and filtering structure changes. Combined phaco-VC is a potent surgery; however, in this study, the contribution of each procedure in achieving successful IOP reduction is not clear. In fact, this study couldn't clarify the exact role of each component of surgery (VC/DS/phaco) in IOP reduction. Additionally, other AS-OCT findings, such as conjunctival changes, choroidal effusion, and TDM thickness, were not considered.

##  Financial Support and Sponsorship 

None.

##  Conﬂict ofs Interest 

None.
